# Divide-and-conquer approach to study protein tunnels in long molecular dynamics simulations

**DOI:** 10.1016/j.mex.2022.101968

**Published:** 2022-12-16

**Authors:** Carlos Sequeiros-Borja, Bartlomiej Surpeta, Igor Marchlewski, Jan Brezovsky

**Affiliations:** a International Institute of Molecular and Cell Biology in Warsaw, Warsaw, Poland; b Laboratory of Biomolecular Interactions and Transport, Department of Gene Expression, Institute of Molecular Biology and Biotechnology, Faculty of Biology, Adam Mickiewicz University, Poznan, Poland

**Keywords:** Transport tunnels, Molecular dynamics simulations, Proteins, High-throughput workflow, MD, Molecular Dynamics, GPUs, graphics processing units, RAM, random-access memory, HDD, hard disk drive, *Divide-and-conquer*

## Abstract

Nowadays, molecular dynamics (MD) simulations of proteins with hundreds of thousands of snapshots are commonly produced using modern GPUs. However, due to the abundance of data, analyzing transport tunnels present in the internal voids of these molecules, in all generated snapshots, has become challenging. Here, we propose to combine the usage of CAVER3, the most popular tool for tunnel calculation, and the TransportTools Python3 library into a divide-and-conquer approach to speed up tunnel calculation and reduce the hardware resources required to analyze long MD simulations in detail. By slicing an MD trajectory into smaller pieces and performing a tunnel analysis on these pieces by CAVER3, the runtime and resources are considerably reduced. Next, the TransportTools library merges the smaller pieces and gives an overall view of the tunnel network for the complete trajectory without quality loss.

## Review Highlights

• The divide-and-conquer approach generates tunnel clusters that are equivalent to the ones obtained when the entire trajectory is analyzed directly by CAVER3.
• Using the divide-and-conquer approach, the runtime and RAM required for tunnel analysis are considerably reduced at least fourfold.

## Graphical abstract

**Figure:**
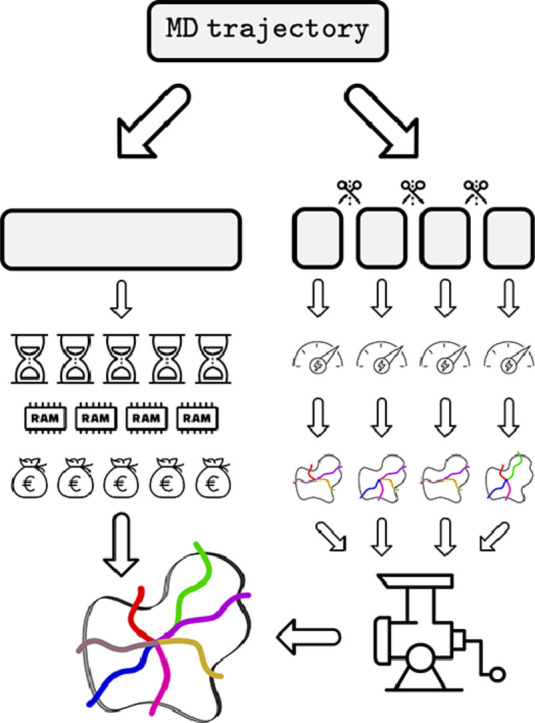

Specifications table

**Table:** 

Subject area:	Bioinformatics
More specific subject area:	*Structural Bioinformatics*
Name of your method:	*Divide-and-conquer*
Name and reference of original method:	*CAVER3 TransportTools*
Resource availability:	https://github.com/labbit-eu/transport_tools
	https://anaconda.org/labbit/transport_tools
	https://pypi.org/project/transport-tools/

## Method details

### Background

Over recent years, computing power has increased and allowed researchers to perform extensive Molecular Dynamics (MD) simulations. Thanks to implementations of MD codes to graphics processing units (GPUs), it is now common to obtain simulations of hundreds of nanoseconds or microseconds without the reliance on specialized hardware or supercomputers [1,2]. However, the difficulty has been transferred to analyzing such a massive dataset. In our case, we focus on the challenges in analyses of internal voids in protein structures, in particular, finding putative transport tunnels. Such tunnels are responsible for transporting ions and small molecules between different regions, connecting inner cavities with the bulk solvent or different cellular environments via transmembrane proteins, making their understanding critical to drug discovery and protein engineering initiatives [3,4]. Although there are several tools available for tunnel analysis in a single structure [5], [6], [7], [8], the users’ options become much more limited when several thousands of structures (snapshots or frames) are considered. Even the most popular tool for tunnel analysis, CAVER3 [5], requires powerful hardware when analyzing extensive data, since, depending on the probe radii employed for the detection of tunnels, it is not uncommon to have tens or hundreds of tunnels per single snapshot.

When faced with an extensively long MD trajectory, above tens of thousands of frames, the user often needs to make compromises to reduce computation time and resources required for tunnel analyses by CAVER3, either (i) only a subset of the whole trajectory, typically every 10th snapshot, is considered, at the cost of less complete tracing of gated transient tunnels, or (ii) the amount of data can be decreased by increasing the probe radius, thereby focusing on rather wide-open tunnels only, which, however, also leads to the loss of data on narrow tunnels and less representative description of close states of wider tunnels too. In case such compromises are not acceptable, the only way is to employ computing resources with tens or even hundreds of gigabytes (GB) of random-access memory (RAM), or make use of algorithms capable of offloading data to the hard disk drive (HDD), considerably limiting the execution times by the input/output (I/O) operations.

Hence, here we propose an alternative approach to analyze tunnels in extensive MD trajectories using a divide-and-conquer approach. We show that slicing the complete trajectory into smaller pieces, then calculating the tunnels with CAVER3 to finally merge these results with the TransportTools library [9] yields outcomes equivalent to those obtained employing the complete trajectory, albeit with reduced hardware requirements and faster calculation time.

### Tunnel calculation

For the method's illustration, the 100 ns long MD simulation of haloalkane dehalogenase DhaA (PDB: 4E46) was used; the details of the computational protocol are available in the Supplementary Protocol. The analyzed trajectory contains 10,000 snapshots. The tunnels present in the protein were identified with CAVER v3.02 [5]. The calculation settings for the whole trajectory and the slices were identical, changing only the last_frame parameter. The starting_point_atom for tunnel search was set as the center of geometry of atoms CG from Asp 106, CD2 from Trp 107, N from Phe 168, and N from Leu 246 (corresponding to PDB: 4E46). For the definition of the starting point, we employed atoms with root mean square fluctuations < 1 Å, whose centroid was located in the deepest part of the active site cavity. The probe_radius was set to 0.7, the clustering_threshold to 3.0, max_output_clusters to 99999, compute_bottleneck_residues to yes, weighting_coefficient to 2, generate_histograms to no, and seed to 1. All the remaining settings were left with default values.

**Fig. 1:**
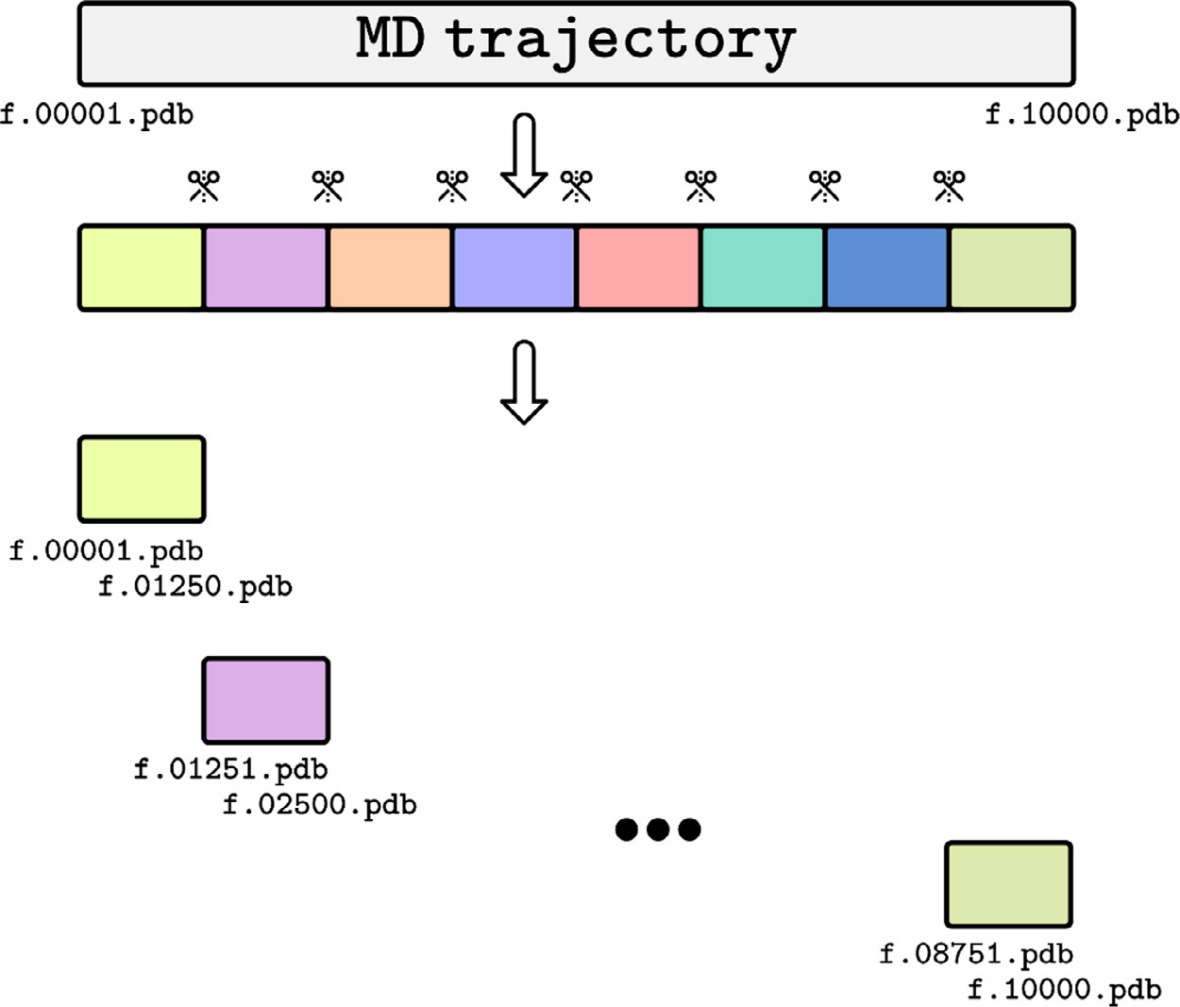
Trajectory slicing for tunnel calculation. Schematic representation of how a long MD trajectory is divided and numbered for the divide-and-conquer approach.

For comparison purposes, the same trajectory was analyzed by two approaches: default processing of the entire trajectory and the divide-and-conquer approach. For the latter, the 10,000 frames were divided into eight pieces of 1,250 frames for each prior tunnel calculation. Although each slice starts technically at frame 1, the numbering of frames was continuous for both approaches, i.e., consecutive slices started with an offset (Fig. 1). This numbering scheme is vital for the procedure to work correctly.

### Merger of tunnel results

**Fig. 2:**
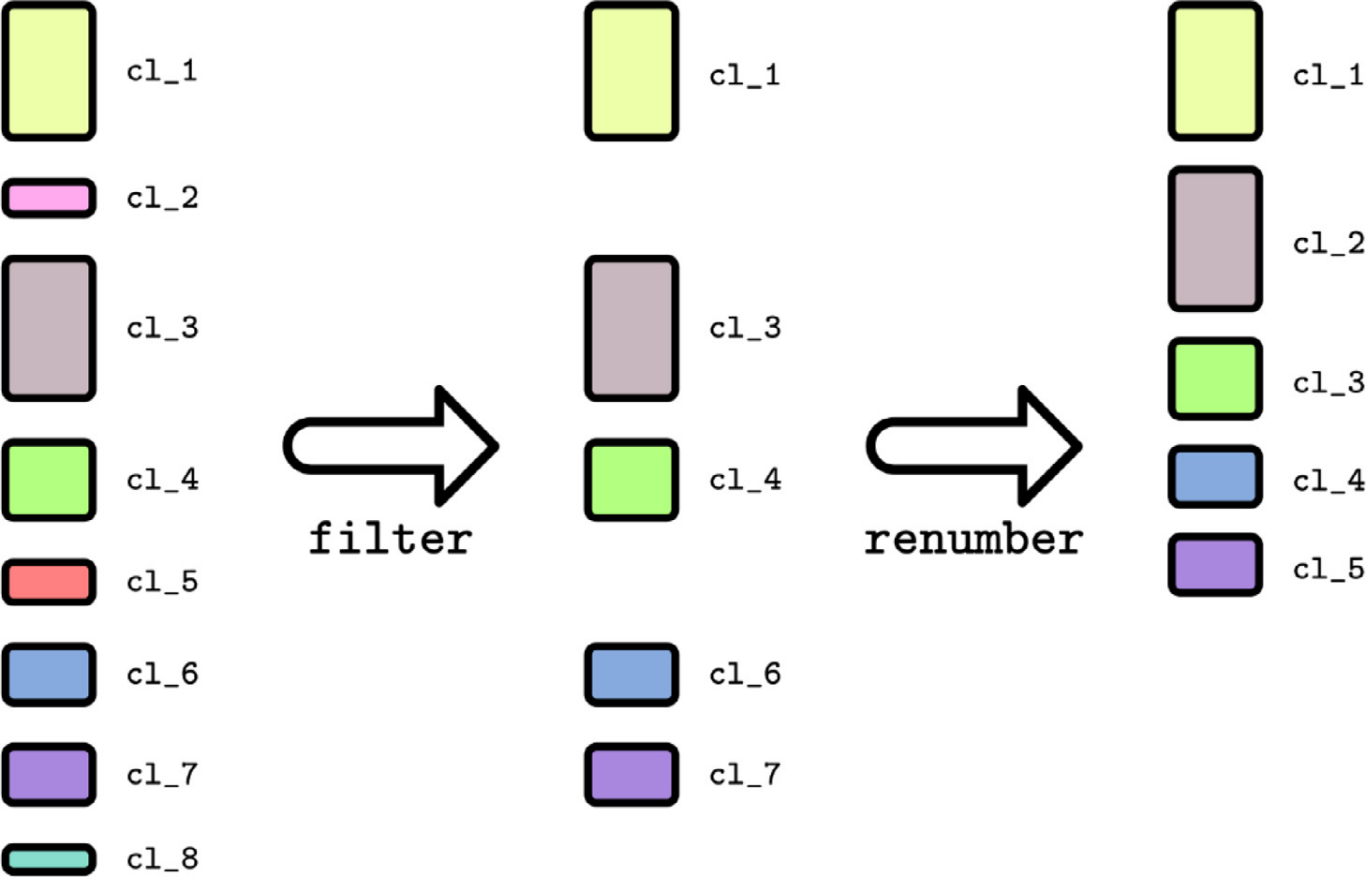
Filtering of CAVER clusters and renumbering. Tunnel clusters that are below a user-defined threshold are removed, then considering the priority of the remaining clusters, a new number ID is given for each cluster.

Once the tunnel calculation from the separate trajectory slices was finished, the results were merged with the TransportTools v0.9.3 library [9]. Although all tunnel clusters calculated from the individual trajectory slices by CAVER3 could, in principle, be included for merging, such an approach significantly increases the subsequent computation time and noise in the final results because the majority of clusters produced contain only a single tunnel instance. Hence, before merging the results, we introduced a filtering stage, in which each tunnel cluster is evaluated on its relative presence in the trajectory slice and excluded if it does not reach a user-defined threshold. We have prepared a Python3 script (*tt_filter_caver_by_frames.py*) that is available from TransportTools v0.9.3 library, to perform this filtering automatically, allowing the user to set the threshold for filtering and if desired, prepare a visualization script of the results in VMD [10] and PyMOL software [11]. During filtering, as stated previously, some clusters were omitted from the final results, which led to numbering inconsistencies if the removed clusters were in between approved clusters (Fig. 2). To overcome this, after the removal, the remaining clusters were renumbered and ranked depending on their priority using the equation:

$$P_{i}=\frac{\sum_{j=1}^{n}T_{j}}{n}$$

**Fig. 3:**
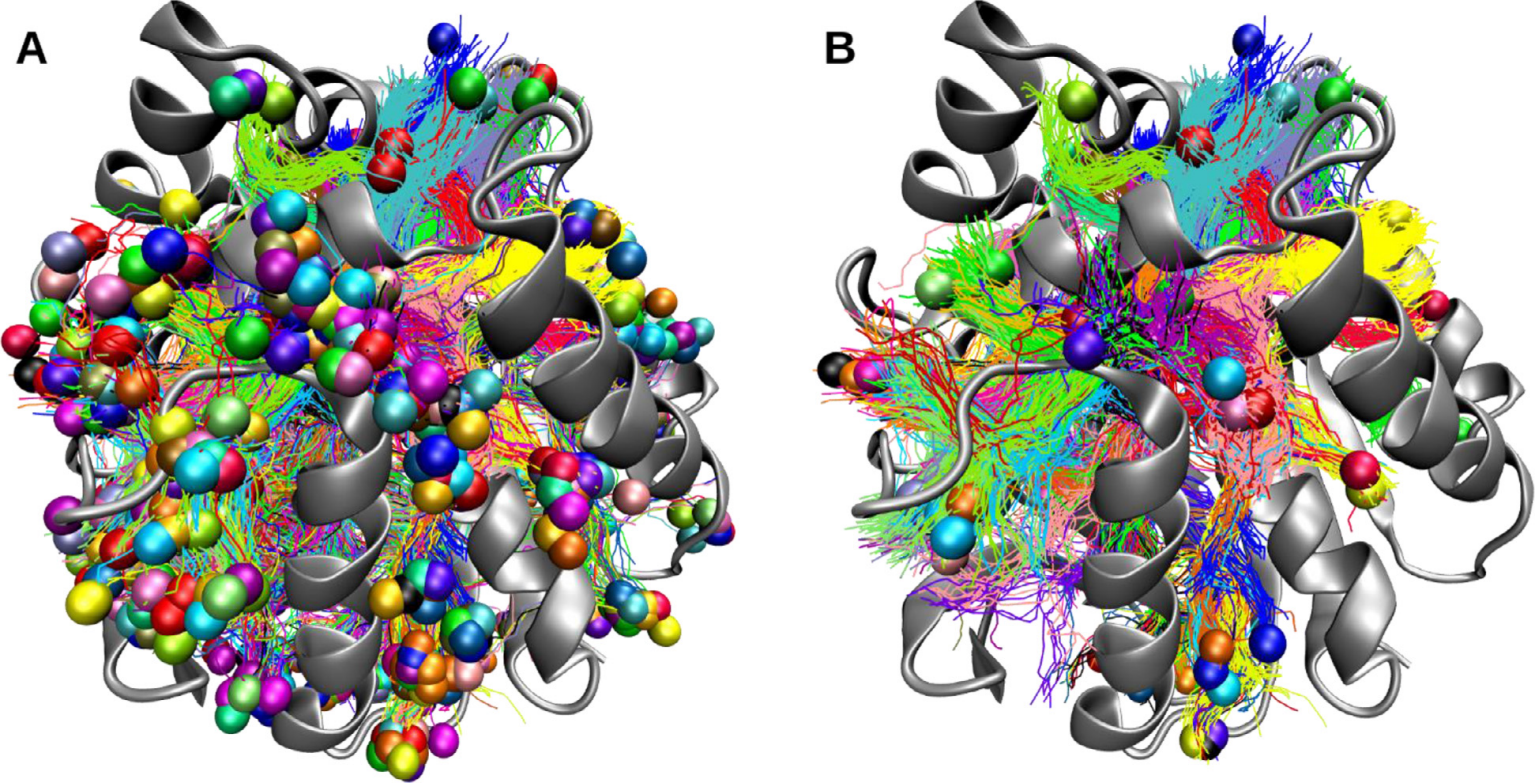
Tunnel clusters present in one trajectory slice of haloalkane dehalogenase. Tunnel clusters were obtained with CAVER3 without filtering (A) and filtering to 2% of presence (B). The protein is represented as a cartoon, tunnels are shown as lines and the last node of each cluster is shown as a sphere.

Where *P_i_* is the priority of cluster *i, T_j_* is the throughput of the tunnel belonging to cluster *i* at frame *j*, and *n* is the total number of frames in the trajectory. If a cluster has no tunnels in a determined frame, its throughput is 0. Although the appropriate threshold for filtering depends on the aims of a particular study and protein system, we have observed that a 2% of presence is an acceptable threshold for the exploration of tunnels. A visual comparison between filtered and non-filtered results is presented in Fig. 3, showcasing the reduction of noise by filtering the results.

With the filtered results, we employed the TransportTools library to merge tunnel clusters from all slices using Ward's clustering method with a cutoff of 1.0 Å. For more information about TransportTools’ capabilities and, in particular, insights into differences among available clustering schemes, the interested reader is encouraged to explore Supplementary Files 2 and 5 in [9]. A significant advantage of employing the TransportTools library for the merging (or clustering) of tunnels from individual trajectory slices is that it can be performed in parallel, considerably decreasing the serial runtime required for CAVER3, even on common workstations with four to eight CPU cores. The resulting superclusters, produced from this divide-and-conquer approach by TransportTools, are analogous to clusters generated by CAVER3, and means for their visualization are provided. Nevertheless, if the CAVER3 output format is desired, we prepared another Python3 script (*tt_convert_to_caver.py*) to convert TransportTools results into CAVER format, including VMD and PyMOL visualization scripts.

### Method validation

**Fig. 4:**
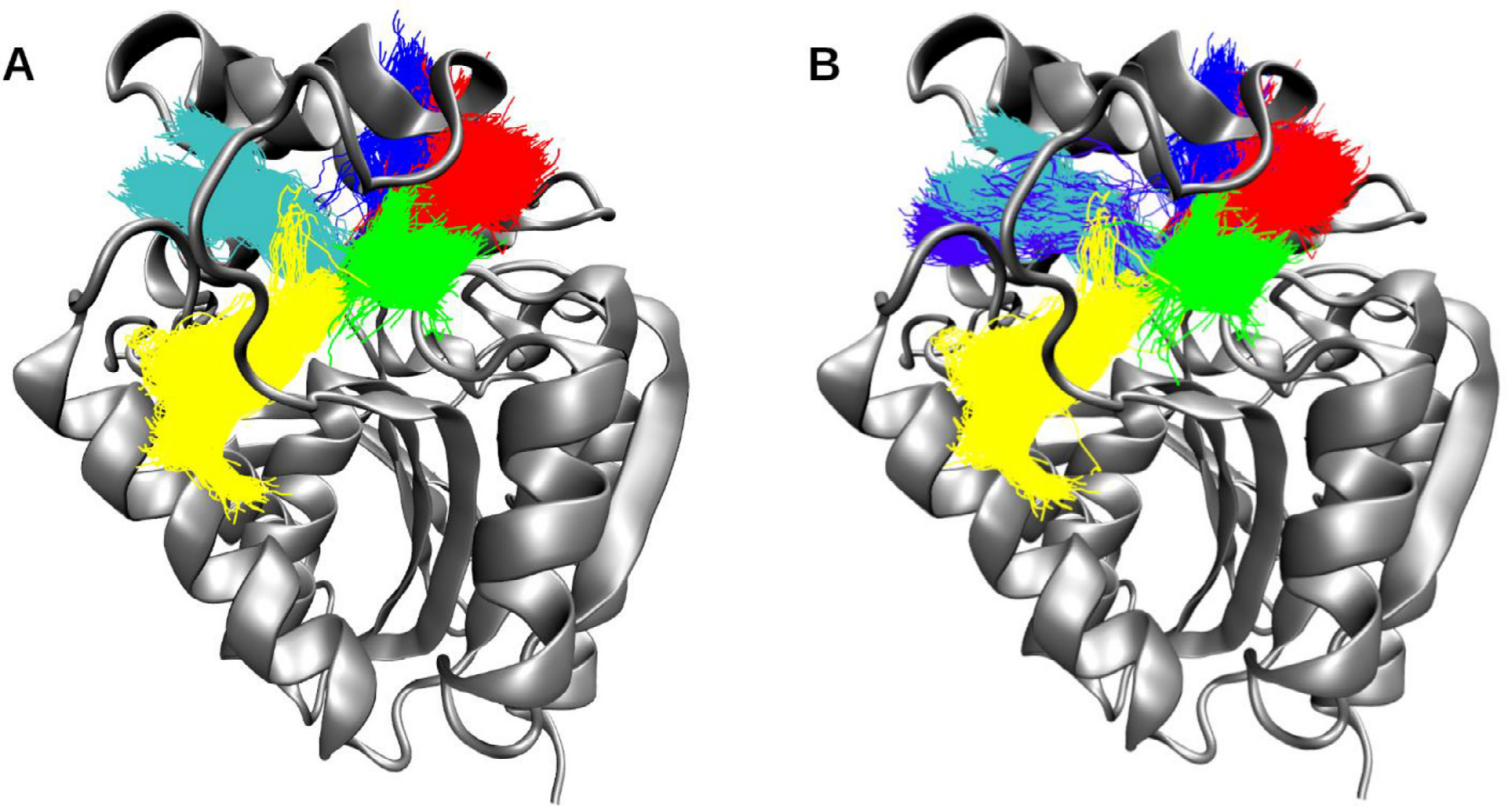
Comparison of top five tunnel clusters. Clusters from the full trajectory generated by CAVER3 (A) and corresponding ones provided by the divide-and-conquer approach (B) are presented. In both approaches, the resulting clusters are ranked equally as seen by the color coding of the clusters, the extra cluster 24 found in the results of divide-and-conquer to also corespond to cluster 4 from the full trajectory analysis is shown in magenta (Table 1).

The results from the full trajectory analysis (Supplementary Fig. 2) were too numerous (above 3,000 tunnel clusters) to perform a detailed comparison with the divide-and-conquer approach. Hence, for simplicity, we will focus on the major clusters with a presence in at least 10% of the entire trajectory, i.e., present in at least 1,000 frames. The CAVER IDs and their corresponding TransportTools supercluster IDs are described in Table 1, and presented in Fig. 4 and Supplementary Fig. 3. The top seven clusters correspond to the same ranking between both approaches, and although from position eight onwards the order is different, all the clusters are present in the divide-and-conquer approach (Table 1 and Supplementary Fig. 3). It is interesting to observe that some CAVER clusters are represented by more than one TransportTools supercluster, *e.g.* clusters with IDs 4, 8, and 12, showcase a more refined separation of the clusters with the proposed approach. The computational resources required to obtain the final results for both approaches are detailed in Table 2. We observed that for the CAVER3 analysis of the full trajectory, the RAM consumption is about five times larger than required for CAVER3 analysis on an trajectory slice used in the divide-and-conquer approach, and can quickly surpass the memory typically available in standard workstations. For example, an analysis of 30,000 frames would require almost 70 GB RAM. Although to make a fair comparison, the divide-and-conquer approach runtime should be multiplied by the number of slices to obtain the total runtime (eight slices in this case), the computation time is still four times shorter than the full trajectory analysis with CAVER3.

**Table 1:** Cluster comparisons of CAVER3 on full trajectory and the divide-and-conquer approach.

CAVER3 on full trajectory					Divide-and-conquer approach				
Cluster ID	Frames	Average bottleneck [Å]	Average length [Å]	Maximal bottleneck [Å]	Cluster ID	Frames	Average bottleneck [Å]	Average length [Å]	Maximal bottleneck [Å]
1	9634	1.098	14.603	2.050	1	9650	1.097	14.622	2.047
2	6658	0.860	16.231	1.530	2	6657	0.860	16.237	1.531
3	4855	0.894	15.845	1.800	3	4791	0.894	15.822	1.798
4	5415	0.790	14.563	1.220	4 / 24	4844 / 1124	0.785 / 0.796	14.437 / 16.282	1.183 / 1.215
5	3877	0.938	18.347	1.390	5	3896	0.937	18.390	1.393
6	2765	0.845	18.313	1.450	6	2739	0.845	18.262	1.453
7	3554	0.797	25.220	1.240	7	3717	0.796	25.568	1.242
8	3817	0.796	24.781	1.210	11 / 14	2686 / 1615	0.793 / 0.795	25.597 / 25.087	1.206 / 1.187
9	2944	0.763	21.388	1.110	8	3135	0.762	21.833	1.109
10	2836	0.771	23.720	1.190	9	2974	0.769	24.105	1.186
11	1362	0.796	16.458	1.520	10	1296	0.792	16.051	1.689
12	2836	0.780	32.352	1.240	20 / 35 / 36	1574 / 1038 / 546	0.777 / 0.762 0.793	34.996 / 33.364 / 29.969	1.242 / 1.021 / 1.091
13	2019	0.772	24.020	1.180	13	2006	0.773	23.976	1.184
14	1543	0.776	24.985	1.090	12	1539	0.774	25.170	1.092
15	1229	0.756	24.481	1.070	17	1161	0.757	24.501	1.074
16	1660	0.759	36.903	1.080	16	1663	0.759	36.952	1.080
17	1492	0.752	33.926	1.030	18	1495	0.753	33.814	1.027
18	1267	0.775	33.571	1.140	22	1224	0.777	33.384	1.138
19	1031	0.764	32.243	1.080	25	1009	0.765	32.223	1.084
20	1491	0.730	33.217	0.910	23	1437	0.730	33.178	0.913
21	1362	0.730	46.357	0.910	43	1265	0.729	45.865	0.907

**Table 2:** Computational resources employed.

	CAVER3 on full trajectory	Divide-and-conquer approach		CAVER3 on reduced trajectory with sparsity 10
		CAVER3 on a trajectory slice a	Merging with TransportTools b	
**Runtime [minutes]**	1,742	57	10	38
**RAM [GB]**	15.4	3.3	2.5	2.5

a The timing is an average of eight CAVER3 calculations, each of 1,250 frames

b Only one TransportTools run is required for the divide-and-conquer approach

As mentioned previously, a common approach to reducing the computational load during tunnel analysis is to take a subset of the original trajectory. Therefore, we also compared the results of tunnel analyses by CAVER3 on a reduced dataset with the sparsity of every 10 snapshots to demonstrate the superior utility of the divide-and-conquer approach. In this case, the top seven clusters correspond to each other between both approaches (Supplementary Table 1 and Supplementary Fig. 4), with the remaining clusters present as well in the divide-and-conquer approach. Additionally, it is visible that the averages for bottleneck radius and tunnel length were comparable in both approaches. Notably, we could observe a systematic underestimation of the maximum bottleneck radius, even up to 0.7 Å (see the highlighted row in Supplementary Table 1), in clusters analyzed from the reduced dataset, which often missed the snapshots with the most open tunnel geometry. As a consequence, the capability of these tunnels for transport would be incorrectly assessed.

## Conclusions

Here, we have presented a new approach for tunnel analysis in long MD trajectories with markedly reduced computational resources and runtimes. We have shown that the results of this approach are equivalent to those obtained in a standard way. We believe that the application of this method will improve the level of detail inferred from tunnel analysis of massive MD data by removing the need for the use of sparse datasets or limiting the detection to only wider tunnels. Lastly, the introduction of this approach makes tunnel analyses on molecular trajectories accessible to researchers lacking powerful computing resources. A detailed guided example of the usage of the divide-and-conquer approach is provided in the Supplementary material.

## CRediT authorship contribution statement

**Carlos Sequeiros-Borja:** Conceptualization, Methodology, Software, Investigation, Writing – original draft, Writing – review & editing, Visualization. **Bartlomiej Surpeta:** Conceptualization, Writing – review & editing. **Igor Marchlewski:** Validation, Formal analysis. **Jan Brezovsky:** Conceptualization, Writing – review & editing, Supervision, Project administration, Funding acquisition.

## Declaration of Competing Interest

The authors declare that they have no known competing financial interests or personal relationships that could have appeared to influence the work reported in this paper.

## Data Availability

The simulated trajectory, standard outputs from analyses of the full trajectory by CAVER3 as well as the divide-and-conquer approach are available from https://doi.org/10.5281/zenodo.7234699.

## Appendix Supplementary materials

Image, application 1
