# Supplementary material for: Divide-and-conquer approach to study protein tunnels in long molecular dynamics simulations
Source: MethodsX. 2022 Dec 16;10:101968. doi: 10.1016/j.mex.2022.101968 (PMC9793300; doi:10.1016/j.mex.2022.101968)
Supplement: Supplementary file 1 [file mmc1.docx]

**Article information**

**Article title**

Divide-and-conquer approach to study protein tunnels in long molecular dynamics simulations

**Authors**

Carlos Sequeiros-Borja,^a,b^ Bartlomiej Surpeta,^a,b^ Igor Marchlewski,^a,b^ Jan Brezovsky^a,b^ *

**Affiliations**

a - International Institute of Molecular and Cell Biology in Warsaw, Warsaw, Poland,

b - Laboratory of Biomolecular Interactions and Transport, Department of Gene Expression, Institute of Molecular Biology and Biotechnology, Faculty of Biology, Adam Mickiewicz University, Poznan, Poland

**Corresponding author’s email address and Twitter handle**

jbrezovsky@iimcb.gov.pl, janbre@amu.edu.pl, @JanBrezovsky1

**Supplementary Protocol**

The 3D structure for the haloalkane dehalogenase [1] protein from *Rhodococcus rhodochrous* was obtained from the Protein Data Bank (PDB: 4E46). The structure was processed to remove all non-protein molecules with exception of crystallographic waters, and the protonation state was determined with the H++ 3.0 webserver [2] at pH 8.5. The water molecules were initially placed around the solute using a tandem approach based on the 3D Reference Interaction Site Model theory [3] (3D-RISM) and the Placevent [4] algorithm. All predicted 3D-RISM waters were subsequently combined with crystallographic waters, keeping only the water molecules at least 2 Å away from the protein using EDIAscorer [5], a method to compute electron density for individual atoms in a crystal structure. The resulting system was then processed with the tLeap module of Amber18 package [6]. The protein was placed at the center of a periodic truncated octahedral box, with a distance cutoff of 10 Å away of the edges and solvated with the OPC water model [7]. Na^+^ and Cl^-^ ions were initially added to neutralize the charge of the system, and subsequently to reach a salt concentration of 0.1 M. The molecular dynamics (MD) simulation was performed with the pmemd.cuda module of Amber18 [8], employing the ff14SB [9] force field. Finally, the Hydrogen Mass Repartitioning method [10] (HMR) was applied to the topology to allow a simulation timestep of 4 fs.

The system was subjected to five minimization cycles with a stepwise release of positional restraints of the protein atoms. The minimization cycles consisted on 100 steps of steepest descent minimization algorithm, followed by 400 steps of conjugated gradient. In the first minimization cycle, harmonic positional restraints were imposed to all heavy atoms of the protein with a force constant of 500 kcal·mol^-1^·Å^2^, and in subsequent cycles, restrictions were applied only to the backbone atoms with force constants of 500, 125, 25, and 0.0001 kcal·mol^-1^·Å^2^ sequentially. After the minimization workflow, a short heating round in the canonical NVT ensemble was applied while keeping the protein’s heavy atoms restrained with a force constant of 5 kcal·mol^-1^·Å^2^. The heating cycle consisted in 20 ps of MD from 0 to 200 K using the Langevin thermostat with a collision frequency of 2 ps^-1^, a coupling constant of 1 ps and simulation timestep of 4 fs. The long-range electrostatic interactions were computed using the Particle Mesh Ewald summation [11] (PME) and all bonds involving hydrogen atoms were constrained using the SHAKE [12] algorithm. Following, four cycles of equilibration were performed in canonical and isobaric-isothermal ensembles. First, in the NVT ensemble, the temperature was raised to the target value of 310 K in 100 ps, employing the same Langevin parameters as previously described. The temperature was kept constant for 900 ps and during the whole procedure, harmonic positional restraints were applied to the protein’s heavy atoms with a force constant of 5 kcal·mol^-1^·Å^2^. Next, 1 ns of MD was run in the NPT ensemble, controlling pressure with the weak-coupling Berendsen barostat with a coupling constant of 1 ps and positional restrains were applied only to backbone atoms using the same force constant as previous stage. Next, another 1 ns of MD in the NPT ensemble with the same setting was performed without any restraints. Final equilibration step constituted a 200 ns simulation with the same settings as the previous step. This was followed by 100 ns of unrestrained production simulation in the canonical NVT ensemble, with constant temperature 310 K and saving frequency of 10 ps. Finally, all water molecules and ions were stripped out, and the trajectory was RMSD-aligned to the first frame using only the backbone atoms (**Supplementary Fig. 1**). For the tunnels analysis, each frame was extracted in PDB file format with the cpptraj [13] module of Amber18.


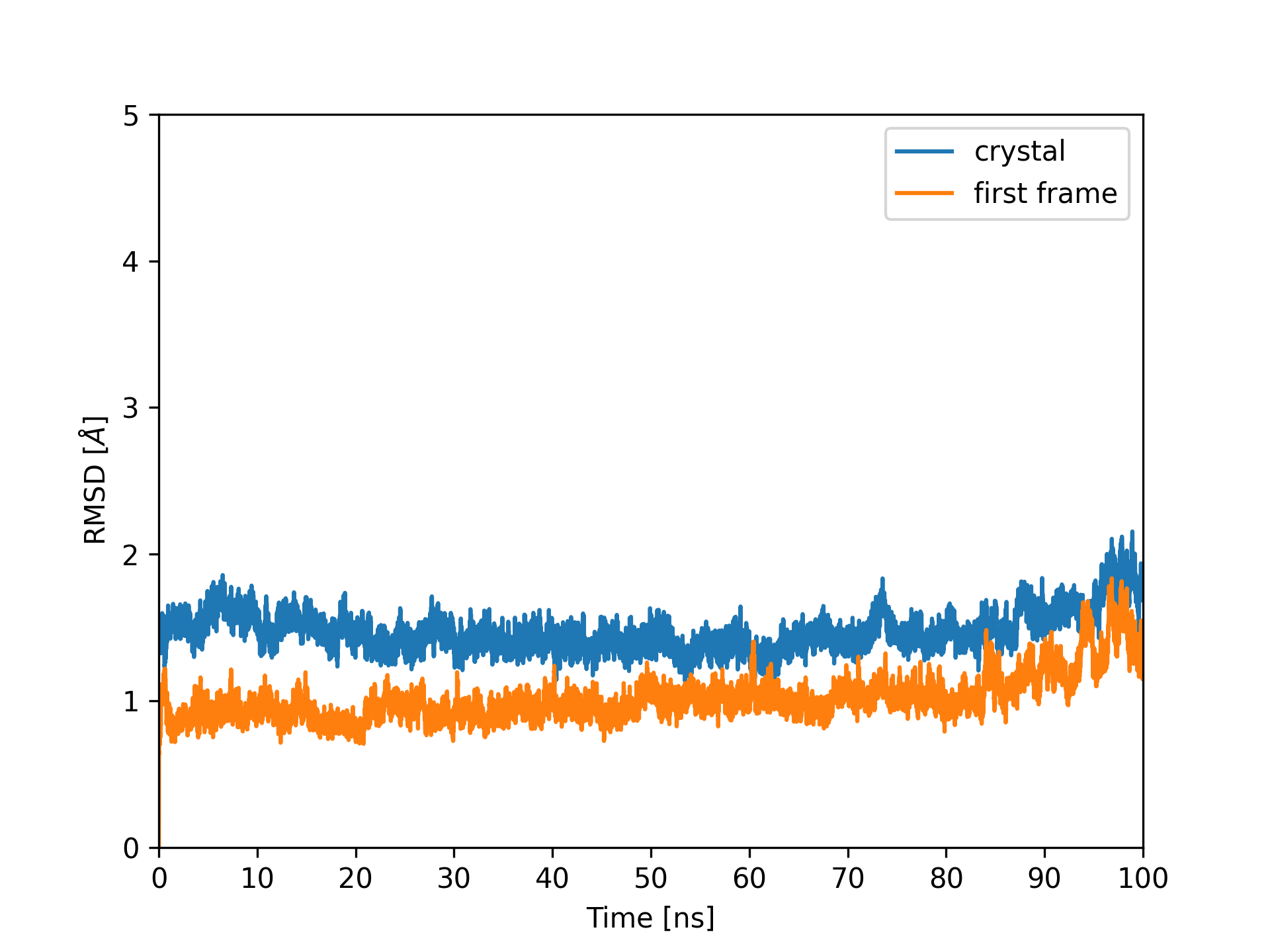


**Supplementary Fig. 1. Backbone root mean square deviation of DhaA.** RMSD plot of the production stage employed for the divide-and-conquer approach. The backbone RMSD to the crystal structure (PDB: 4E46), and the first frame of the simulation are shown in blue and orange, respectively.

**Supplementary References**

[1] V. Stepankova, M. Khabiri, J. Brezovsky, A. Pavelka, J. Sykora, M. Amaro, B. Minofar, Z. Prokop, M. Hof, R. Ettrich, R. Chaloupkova, J. Damborsky, Expansion of Access Tunnels and Active-Site Cavities Influence Activity of Haloalkane Dehalogenases in Organic Cosolvents, ChemBioChem. 14 (2013) 890–897. https://doi.org/10.1002/cbic.201200733.

[2] R. Anandakrishnan, B. Aguilar, A. V. Onufriev, H++ 3.0: Automating pK prediction and the preparation of biomolecular structures for atomistic molecular modeling and simulations, Nucleic Acids Res. 40 (2012) W537–W541. https://doi.org/10.1093/nar/gks375.

[3] T. Luchko, S. Gusarov, D.R. Roe, C. Simmerling, D.A. Case, J. Tuszynski, A. Kovalenko, Three-dimensional molecular theory of solvation coupled with molecular dynamics in amber, J. Chem. Theory Comput. 6 (2010) 607–624. https://doi.org/10.1021/ct900460m.

[4] D.J. Sindhikara, N. Yoshida, F. Hirata, Placevent: An algorithm for prediction of explicit solvent atom distribution-Application to HIV-1 protease and F-ATP synthase, J. Comput. Chem. 33 (2012) 1536–1543. https://doi.org/10.1002/jcc.22984.

[5] A. Meyder, E. Nittinger, G. Lange, R. Klein, M. Rarey, Estimating Electron Density Support for Individual Atoms and Molecular Fragments in X-ray Structures, J. Chem. Inf. Model. 57 (2017) 2437–2447. https://doi.org/10.1021/acs.jcim.7b00391.

[6] D.A. Case, I.Y. Ben-Shalom, S.R. Brozel, D.S. Cerutti, T.E. Cheatham, V.W.D. Cruzeiro, T.A. Darden, R.E. Duke, D. Ghoreishi, M.K. Gilson, H. Gohlke, A.W. Goetz, D. Greene, R. Harris, N. Homeyer, Y. Huang, S. Izadi, A. Kovalenko, T. Kurtzman, T.S. Lee, S. LeGrand, P. Li, C. Lin, J. Liu, T. Luchko, R. Luo, D.J. Mermelstein, K.M. Merz, Y. Miao, G. Monard, C. Nguyen, H. Nguyen, I. Omelyan, A. Onufriev, F. Pan, R. Qi, D.R. Roe, A. Roitberg, C. Sagui, S. Schott-Verdugo, J. Shen, C.L. Simmerling, J. Smith, R. Salomon-Ferrer, J. Swails, R.C. Walker, J. Wang, H. Wei, R.M. Wolf, X. Wu, L. Xiao, D.M. York, P.A. Kollman, AMBER 18, (2018). ambermd.org.

[7] S. Izadi, R. Anandakrishnan, A. V. Onufriev, Building water models: A different approach, J. Phys. Chem. Lett. 5 (2014) 3863–3871. https://doi.org/10.1021/jz501780a.

[8] R. Salomon-Ferrer, A.W. Götz, D. Poole, S. Le Grand, R.C. Walker, Routine microsecond molecular dynamics simulations with AMBER on GPUs. 2. Explicit solvent particle mesh ewald, J. Chem. Theory Comput. 9 (2013) 3878–3888. https://doi.org/10.1021/ct400314y.

[9] J.A. Maier, C. Martinez, K. Kasavajhala, L. Wickstrom, K.E. Hauser, C. Simmerling, ff14SB: Improving the Accuracy of Protein Side Chain and Backbone Parameters from ff99SB, J. Chem. Theory Comput. 11 (2015) 3696–3713. https://doi.org/10.1021/acs.jctc.5b00255.

[10] C.W. Hopkins, S. Le Grand, R.C. Walker, A.E. Roitberg, Long-time-step molecular dynamics through hydrogen mass repartitioning, J. Chem. Theory Comput. 11 (2015) 1864–1874. https://doi.org/10.1021/ct5010406.

[11] T. Darden, D. York, L. Pedersen, Particle mesh Ewald: An N·log(N) method for Ewald sums in large systems, J. Chem. Phys. 98 (1993) 10089–10092. https://doi.org/10.1063/1.464397.

[12] J.P. Ryckaert, G. Ciccotti, H.J.C. Berendsen, Numerical integration of the cartesian equations of motion of a system with constraints: molecular dynamics of n-alkanes, J. Comput. Phys. 23 (1977) 327–341. https://doi.org/10.1016/0021-9991(77)90098-5.

[13] D.R. Roe, T.E. Cheatham, PTRAJ and CPPTRAJ: Software for processing and analysis of molecular dynamics trajectory data, J. Chem. Theory Comput. 9 (2013) 3084–3095. https://doi.org/10.1021/ct400341p.


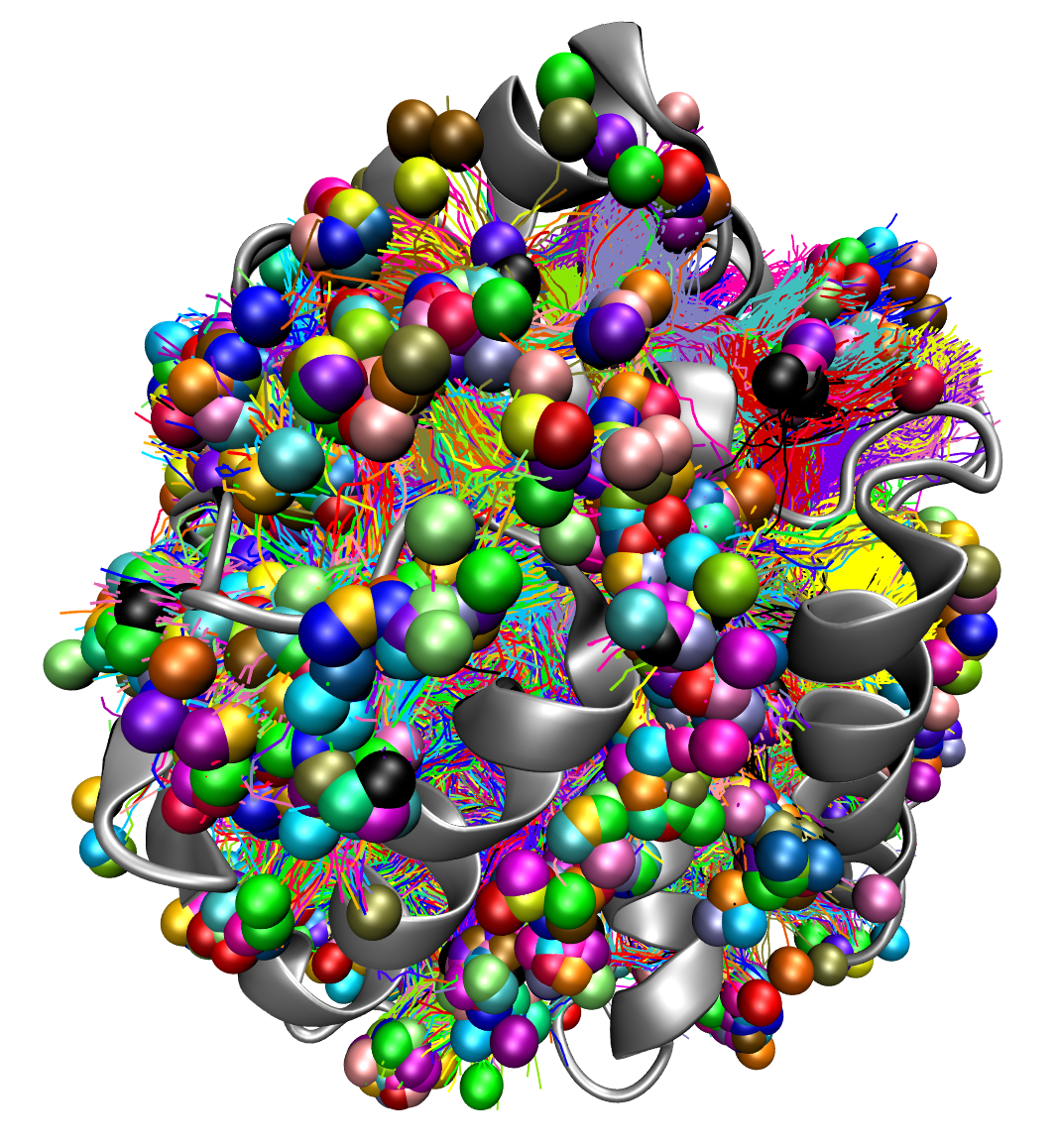


**Supplementary Fig. 2.** **CAVER3 results from the complete MD trajectory.** All tunnel clusters generated for 10,000 frames are presented as lines with the protein represented as cartoon and the last node of each cluster shown as sphere.


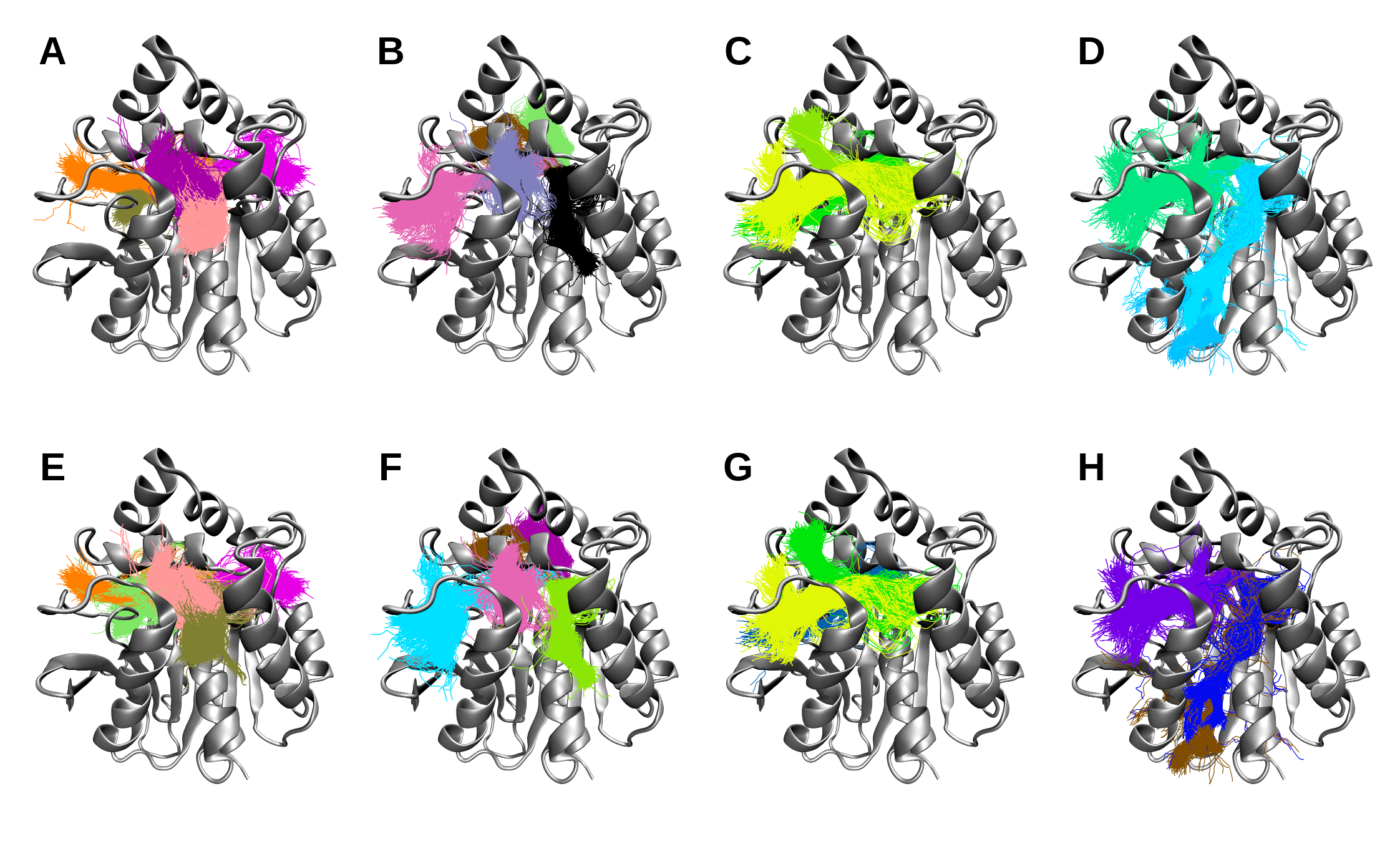
**Supplementary Fig. 3.** **Tunnel clusters obtained from full trajectory analysis by CAVER3 and the divide-and-conquer approaches.** Tunnel clusters coming from the full trajectory analysis by CAVER3 (A-D) and the divide-and-conquer approach (E-H). Cluster ranked 6-10 (A), ranked 11-15 (B), ranks 16-18 (C) and ranks 19-21 (D) for full trajectory and their corresponding clusters from the divide-and-conquer approach according to Table 1 are presented. The colors of each cluster correspond to their rank IDs, hence the difference in the clusters colors from both approaches.


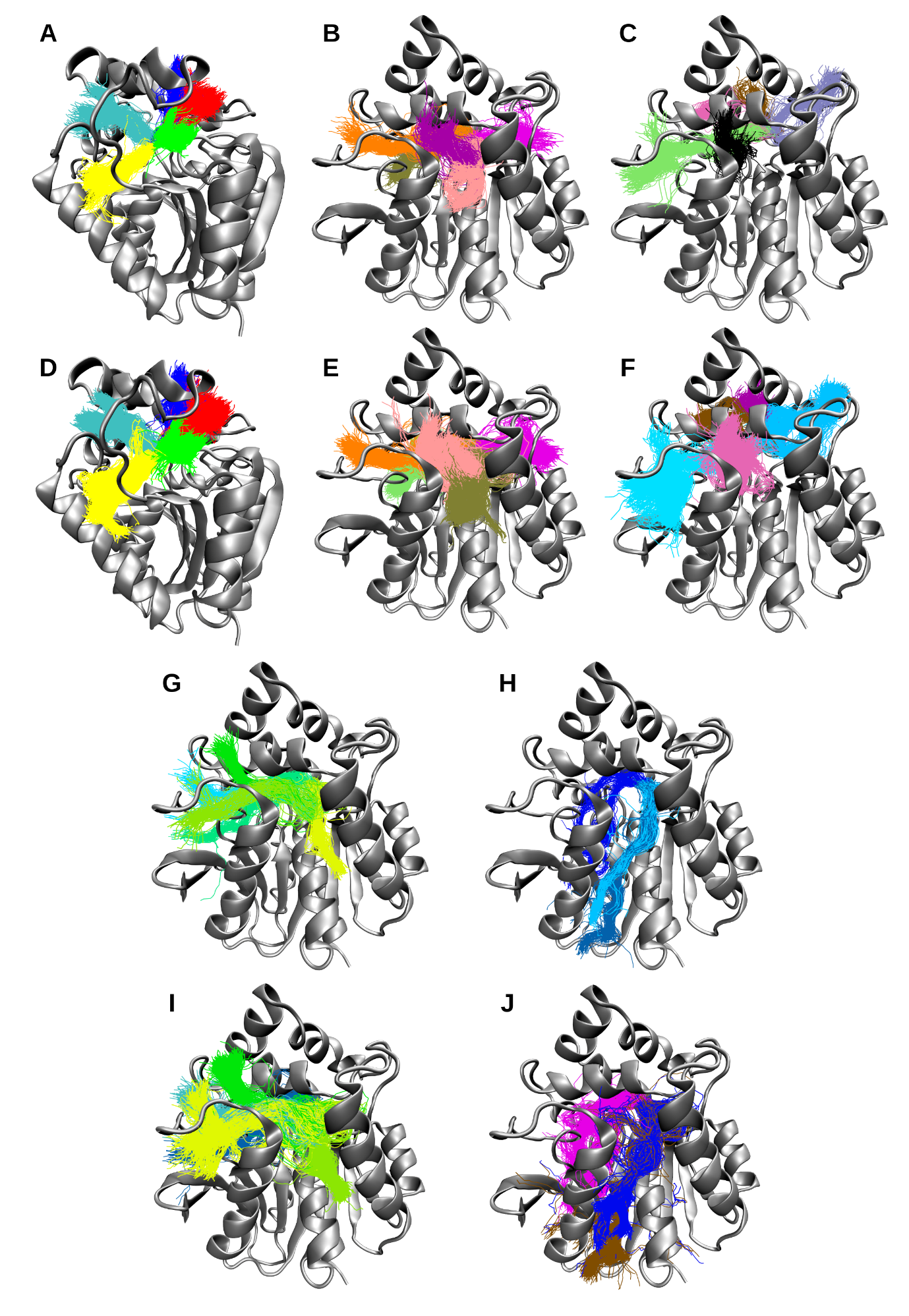
**Supplementary Fig. 4.** **Tunnel clusters obtained from reduced dataset analyzed by CAVER3 with sparsity and the divide-and-conquer approaches.** Tunnel clusters coming from the sparsity analysis (A-C, G-H) and the divide-and-conquer approach (D-F, I-J) are shown side by side. Cluster ranked 1-5 (A), ranks 6-10 (B), ranks 11-15 (C), ranks 16-19 (G) and ranks 20-23 (D) for sparsity trajectory and their corresponding clusters from the divide-and-conquer approach according to Supplementary Table 2 are presented. The colors of each cluster correspond to their rank IDs, hence the difference in the clusters colors from both approaches.

| **Supplementary Table 1. Cluster comparisons of CAVER3 on reduced trajectory with sparsity 10, and the divide-and-conquer approach.** | | | | | | | | | |
| --- | --- | --- | --- | --- | --- | --- | --- | --- | --- |
| **CAVER3 on reduced trajectory with sparsity 10** | | | | | **Divide-and-conquer approach** | | | | |
| **Cluster ID** | **Frames** | **Average bottleneck [Å]** | **Average length [Å]** | **Maximal bottleneck [Å]** | **Cluster ID** | **Frames** | **Average bottleneck [Å]** | **Average length [Å]** | **Maximal bottleneck [Å]** |
| 1 | 950 | 1.098 | 14.578 | 1.790 | 1 | 9650 | 1.097 | 14.622 | 2.047 |
| 2 | 680 | 0.859 | 16.333 | 1.340 | 2 | 6657 | 0.860 | 16.237 | 1.531 |
| 3 | 480 | 0.886 | 15.842 | 1.410 | 3 | 4791 | 0.894 | 15.822 | 1.798 |
| 4 | 523 | 0.784 | 14.611 | 1.170 | 4 | 4844 | 0.785 | 14.437 | 1.183 |
| 5 | 386 | 0.939 | 18.389 | 1.330 | 5 | 3896 | 0.937 | 18.390 | 1.393 |
| 6 | 273 | 0.846 | 18.641 | 1.210 | 6 | 2739 | 0.845 | 18.262 | 1.453 |
| 7 | 343 | 0.796 | 25.501 | 1.110 | 7 | 3717 | 0.796 | 25.568 | 1.242 |
| 8 | 343 | 0.797 | 24.940 | 1.090 | 11 | 2686 | 0.793 | 25.597 | 1.206 |
| 9 | 293 | 0.768 | 21.501 | 1.110 | 8 | 3135 | 0.762 | 21.833 | 1.109 |
| 10 | 281 | 0.774 | 23.928 | 1.100 | 9 | 2974 | 0.769 | 24.105 | 1.186 |
| 11 | 292 | 0.778 | 32.271 | 1.090 | 20 | 1574 | 0.777 | 34.996 | 1.242 |
| 12 | 204 | 0.779 | 23.877 | 1.130 | 13 | 2006 | 0.773 | 23.976 | 1.184 |
| 13 | 116 | 0.758 | 16.265 | 0.990 | 10 | 1296 | 0.792 | 16.051 | 1.689 |
| 14 | 130 | 0.774 | 18.698 | 1.090 | 21 | 875 | 0.783 | 18.736 | 1.124 |
| 15 | 152 | 0.773 | 25.073 | 1.040 | 12 | 1539 | 0.774 | 25.170 | 1.092 |
| 16 | 124 | 0.760 | 24.451 | 0.930 | 17 | 1161 | 0.757 | 24.501 | 1.074 |
| 17 | 173 | 0.758 | 37.246 | 1.010 | 16 | 1663 | 0.759 | 36.952 | 1.080 |
| 18 | 155 | 0.757 | 34.110 | 0.950 | 18 | 1495 | 0.753 | 33.814 | 1.027 |
| 19 | 108 | 0.766 | 34.606 | 1.090 | 22 | 1224 | 0.777 | 33.384 | 1.138 |
| 20 | 105 | 0.759 | 42.072 | 0.950 | 34 | 771 | 0.762 | 42.058 | 1.080 |
| 21 | 111 | 0.732 | 34.580 | 0.870 | 23 | 1437 | 0.730 | 33.178 | 0.913 |
| 22 | 141 | 0.732 | 46.442 | 0.840 | 43 | 1265 | 0.729 | 45.865 | 0.907 |
| 23 | 101 | 0.739 | 46.050 | 0.860 | 53 / 54 | 551 / 565 | 0.737 / 0.735 | 43.129 / 47.656 | 0.912 / 0.873 |

**Guided Example**

To use the divide-and-conquer approach with *TransportTools*, at least version 0.9.3 of the library must be installed. Install instructions are present at the github repository [[github](https://github.com/labbit-eu/transport_tools)]. The MD data and scripts necessary to run this guided example can be downloaded from the repository at Zenodo [[zenodo](https://doi.org/10.5281/zenodo.7234699)]. For this tutorial, we assume the user is in a UNIX environment.

1. **Generate split inputs into batches and run tunnel calculation separately**

Download 05_guided_example.tar.gz from this paper repository [[zenodo](https://doi.org/10.5281/zenodo.7234699)] and extract the content. Open a terminal and enter *05_guided_example/01_inputs* directory.

cd 05_guided_example/01_inputs

You should see following content (**Supplementary Fig. 5**):


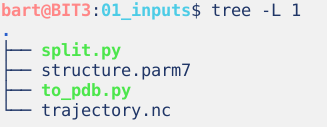


**Supplementary Fig. 5.** Input files and scripts necessary to prepare PDB ensembles for CAVER3 calculations.

At the initial stage, the input trajectory has to be converted into the ensemble of PDB files required for CAVER calculations. For this purpose, we will use the *mdtraj* package already installed in the *TransportTools* environment. To generate PDB files execute the command:

python3 to_pdb.py

Successful conversion of the input trajectory should result in the following information (**Supplementary Fig. 6**):


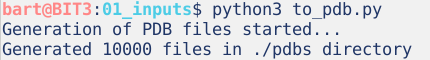


**Supplementary Fig. 6.** Output information confirming successful conversion of *.netcdf trajectory to set of PDB files.

Next, we use *split.py* script to split PDB files into eight batches of 1,250 frames each:

python3 split.py

Execution of the command above will generate eight directories part_1 to part_8.

In the case you plan to perform the CAVER calculation yourself, enter *02_sliced_trajectory* directory and move all *part_** directories from *01_inputs* containing PDB files there. In this directory, execute *01_prepare_cavers.sh* to create the configuration file for each split, and then execute *02_run_cavers.sh* script to perform CAVER calculations.

bash 01_preprare_cavers.sh

bash 02_run_cavers.sh

Note: In case you want to use precomputed CAVER split results, copy them from 02_sliced_trajectory.tar.gz [[here](https://zenodo.org/record/7234700/files/02_sliced_trajectory.tar.gz?download=1)] file in a repository and continue to the next section.

1. **Merge tunnels calculated in batches and filter them**

Now, we will focus on the key point of this section. Enter *03_sliced_filtered* directory. You will find here three bash scripts corresponding to three stages that will be performed, as well as the CAVER reference PDB file and *TransportTools* configuration file (**Supplementary Fig. 7**):


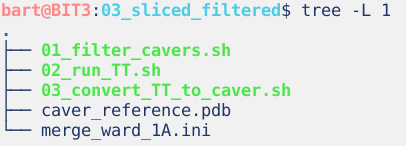


**Supplementary Fig. 7.** Input files and scripts necessary for the merging procedure of divided CAVER calculations using TransportTools library.

In the first stage of the procedure (*01_filter_cavers.sh*), we will step-by-step remove clusters from each *part_** CAVER results that contain fewer tunnels than the specified threshold. For this guided example, we are filtering out clusters that have fewer tunnels than 2% of frames analyzed in each batch (using the flag -l 25, as 2% of 1,250 frames using the flag -f 1250). This will be performed by *tt_filter_caver_by_frames.py* script available in *TransportTools*. The usage of this script can be checked by executing this script with the flag -h, it will provide the following output (**Supplementary Fig. 8**):


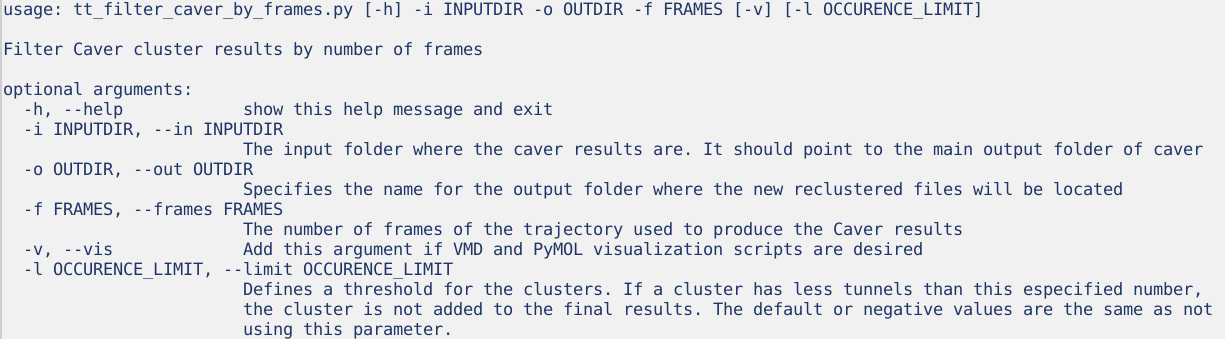


**Supplementary Fig. 8.** Usage of tt_filter_caver_by_frames.py with the available options.

Now, let’s perform the filtering:

bash 01_filter_cavers.sh

Please note that this script generated *filter_part_*.log* file in the directory corresponding to each batch with the information about filtered-out clusters and following clusters’ renumbering (outputting original and new cluster IDs).

Now, we will perform the merging stage using tt_engine.py. Here, we will use *clustering_linkage = ward* with *clustering_cutoff = 1.0*, as it performed best during merging based on thorough testing in our Laboratory.

bash 02_run_TT.py

Note: It takes roughly ~30 minutes, using 4 CPU cores.

As specified in the configuration file, a successful *tt_engine.py* run produces the *ward_1_results* output directory. Here, you should expect standard *TransportTools* output, including *data*, *_internal*, *statistics*, and *visualization* directories. The *transport_tools.log* file will be also generated here. At this point, it is worth highlighting several elements. First, in *data/super_clusters/details/initial_super_cluster_details.txt* one can find details regarding the source data for each supercluster, namely which tunnel clusters from particular *part_** are forming it. At the same time, *bottlenecks* and *CSV_profiles* directories contain information about bottlenecks and tunnel profiles, respectively. Second, in the statistics directory, two output files are generated corresponding to superclusters statistics (*1-initial_tunnels_statistics.txt* which is a CAVER‑summary-like-file) as well as the file listing the bottleneck residues (*1‑initial_tunnels_statistics_bottleneck_residues.txt*). The simplified output tree can be found in **Supplementary Fig. 9**, while the supercluster statistics are in **Supplementary Fig. 10**. Finally, the visualization directory includes a script for the visualization of superclusters in PyMOL.


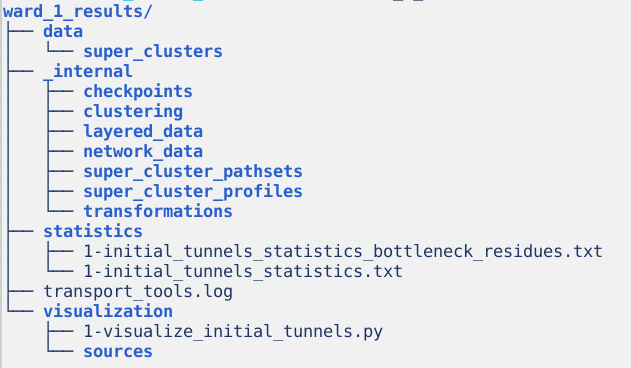


**Supplementary Fig. 9.** Content of the TransportTools merging calculations output directory.

At this stage, all important information analogous to CAVER full trajectory run is available. Nevertheless, if the native CAVER output format is required, we also provide the functionality to convert *TransportTools* merged results to CAVER formatted outputs. This can be performed using the *tt_convert_to_caver.py* script, using the same configuration file as during merging. **Supplementary Fig. 11** presents the usage of this script when executed with the flag -h.


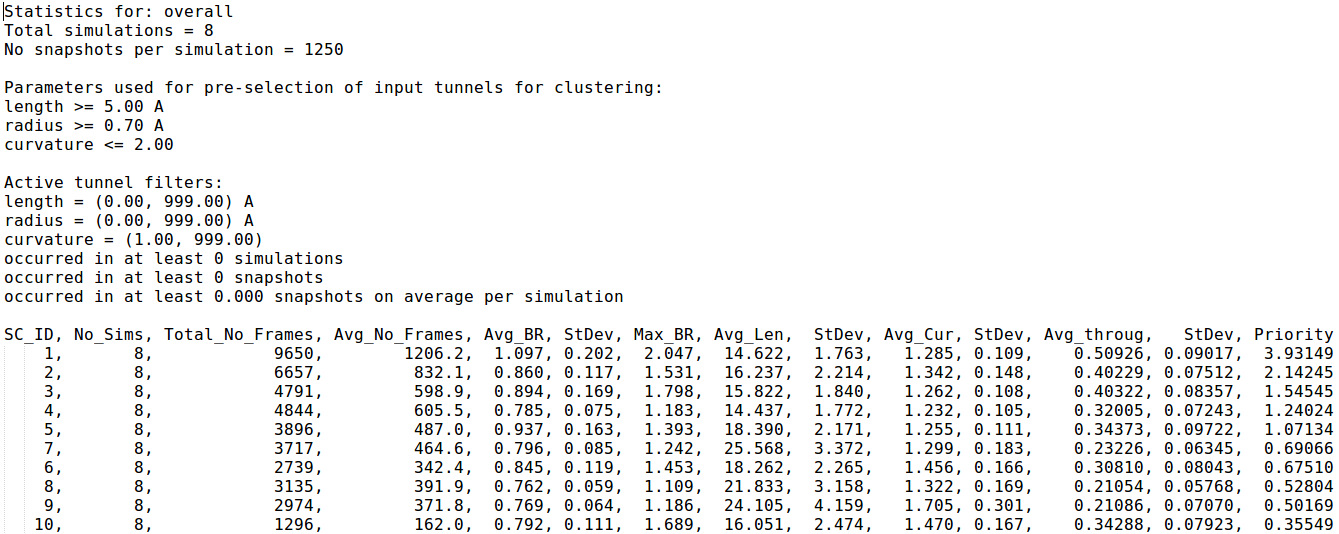


**Supplementary Fig. 10.** TransportTools statistics for the first 10 superclusters obtained in the merging procedure.


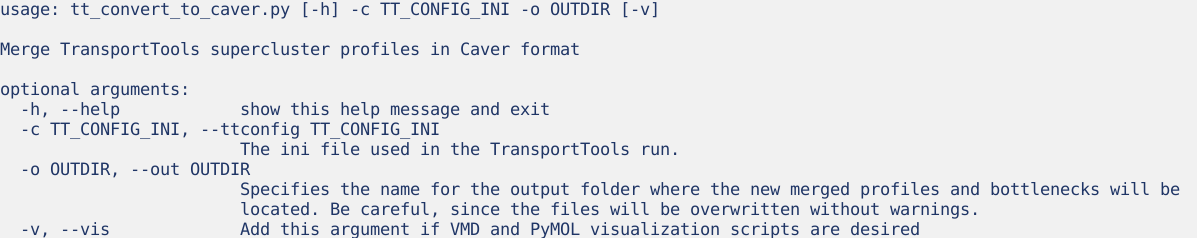


**Supplementary Fig. 11.** Usage of tt_convert_to_caver.py with the available options.

It is encapsulated in 03_convert_TT_to_caver.sh script:

bash 03_convert_to_caver.sh

The successful execution of this script will generate outputs in the folder specified by the -o flag for *tt_convert_to_caver.py*, in our case *divide_and_conquer*. Output directories and files are presented in the form of a simplified tree in **Supplementary Fig. 12**. They include the analysis directory with CAVER-like *bottlenecks.csv* and *tunnel_profiles.csv* files, the *data* folder with *clusters_timeless*, *origins* and representative snapshot, *summary.txt* file, and finally the *vmd* and *pymol* folders with all files necessary for the visualization using VMD and PyMOL software, respectively.


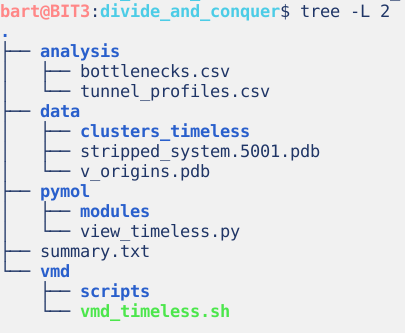


**Supplementary Fig. 12.** Output TransportTools directory converted to the original CAVER output format.
